# Supplementary material for: Exploring the Rumen Microbiota and Serum Metabolite Profile of Hainan Black Goats with Different Body Weights before Weaning
Source: Animals (Basel). 2024 Jan 28;14(3):425. doi: 10.3390/ani14030425 (PMC10854652; doi:10.3390/ani14030425)
Supplement: Supplementary file 1 [file animals-14-00425-s001.zip › Supplemental Table.pdf]

Supplementary File – for Online Publication Only

Supplemental Table S1. Composition and nutrient levels of the basal diet (DM basis).

Ingredients	Mix concentrates	Mix coarse materials
Corn %	25.68	-
Soybean meal %	7.38	-
Peanut seedlings %	7.33	-
Wheat bran %	7.31	-
Premix[1] %	1.0	-
NaCl %	0.73	-
Na_2O_3 %	0.57	-
King grass %	-	22.25
Whole corn %	-	20.37
King grass silage %	-	7.38
Total %	50.0	50.0
Nutrient levels[2]		
Dry matter %	88.63	26.93
Ash %	6.27	7.58
Crude protein %	14.21	3.98
Ether extract %	4.73	3.85
Neutral detergent fibers %	14.33	67.33
Acid detergent fibers %	4.35	45.35
Ca %	0.62	0.81
P %	0.47	0.33
Metabolic energy (MJ/kg)	16.61	10.36

[1] The premix provided the following per kg of the diet: VA 9 000 IU, VD 3 000 IU, VE 50 IU, Cu 15 mg, Fe 700 mg, Mn 500 mg, Zn 800 mg, I 1.0 mg, Se7.0 mg, Co 20 mg, Ca 14 g, P 1.0 g.
[2] Metabolic energy was a calculated value, while the others were measured values.

Supplementary Table S2. Differences in rumen morphology of goat kids with different body weight.

Items	Group[1]		SEM	*P*-value
	HBW	LBW		
Papillae length (μm)	1232.57	656.36	137.15	0.016
Papillae width (μm)	742.06	563.33	47.11	0.042
Muscle layer thickness (μm)	338.53	314.82	38.89	0.564
Rumen weight (g)	120.13	90.24	0.04	0.178

[1]HBW = high body weight; LBW= low body weight.

Supplementary Table S3. The serum biochemical indicators of LBW and HBW goat kids.

Items[1]	Group[2]		SEM	*P*-value
	HBW	LBW		
TP (g/dL)	6.66	6.72	0.19	0.961
Albumin (g/dL)	3.29	3.41	0.21	0.787
Globulin (g/dL)	3.66	3.10	1.58	0.621
BUN (mg/dL)	45.65	49.91	9.73	0.938
Glucose (mg/dL)	59.94	47.52	14.49	0.050
TC (mg/dL)	52.04	44.23	10.21	0.046
TG (U/L)	2.90	2.53	0.29	0.042
LDH (U/L)	345.00	396.41	58.74	0.411
ALT (U/L)	22.81	16.84	7.02	0.424
AST (U/L)	78.62	94.02	15.73	0.352
ALP (U/L)	283.82	289.60	121.30	0.368

[1]TP, total protein; BUN, blood urea nitrogen; TC, total cholesterol; TG, triglyceride; LDH, lactate dehydrogenase; ALT, alanine transaminase; AST, aspartate transaminase; ALP, alkaline phosphatase.

[2]HBW = high body weight; LBW= low body weight.

Supplementary Table S4. Valid operational taxonomic units (OTUs) and α-diversity diversity indices of rumen microbiota in HBW and LBW goat kids.

Items	Group[1]		SEM	*P*-value
	HBW	LBW		
OTUs	4162	2905	208.60	0.042
Chao1	5974.39	4309.08	253.91	0.025
Shannon	31.32	26.16	0.99	0.046
Simpson	4.74	4.47	0.05	0.171

[1]HBW = high body weight; LBW= low body weight.
