# Supplementary figures and images for: Exploring the Rumen Microbiota and Serum Metabolite Profile of Hainan Black Goats with Different Body Weights before Weaning
Source: Animals (Basel). 2024 Jan 28;14(3):425. doi: 10.3390/ani14030425 (PMC10854652; doi:10.3390/ani14030425)

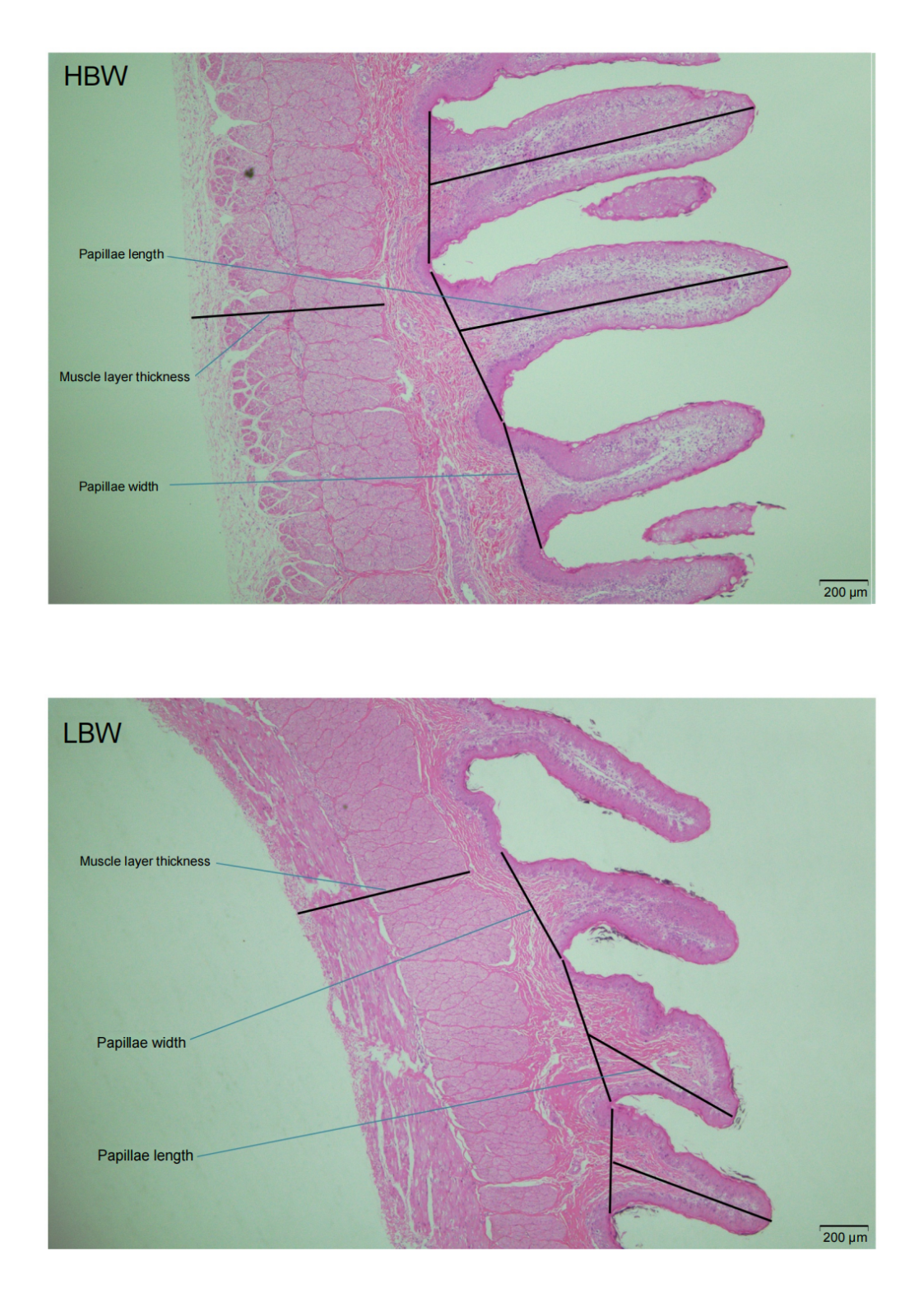

Supplement: Supplementary file 1 [file animals-14-00425-s001.zip › Supplemental Figure S1..png]

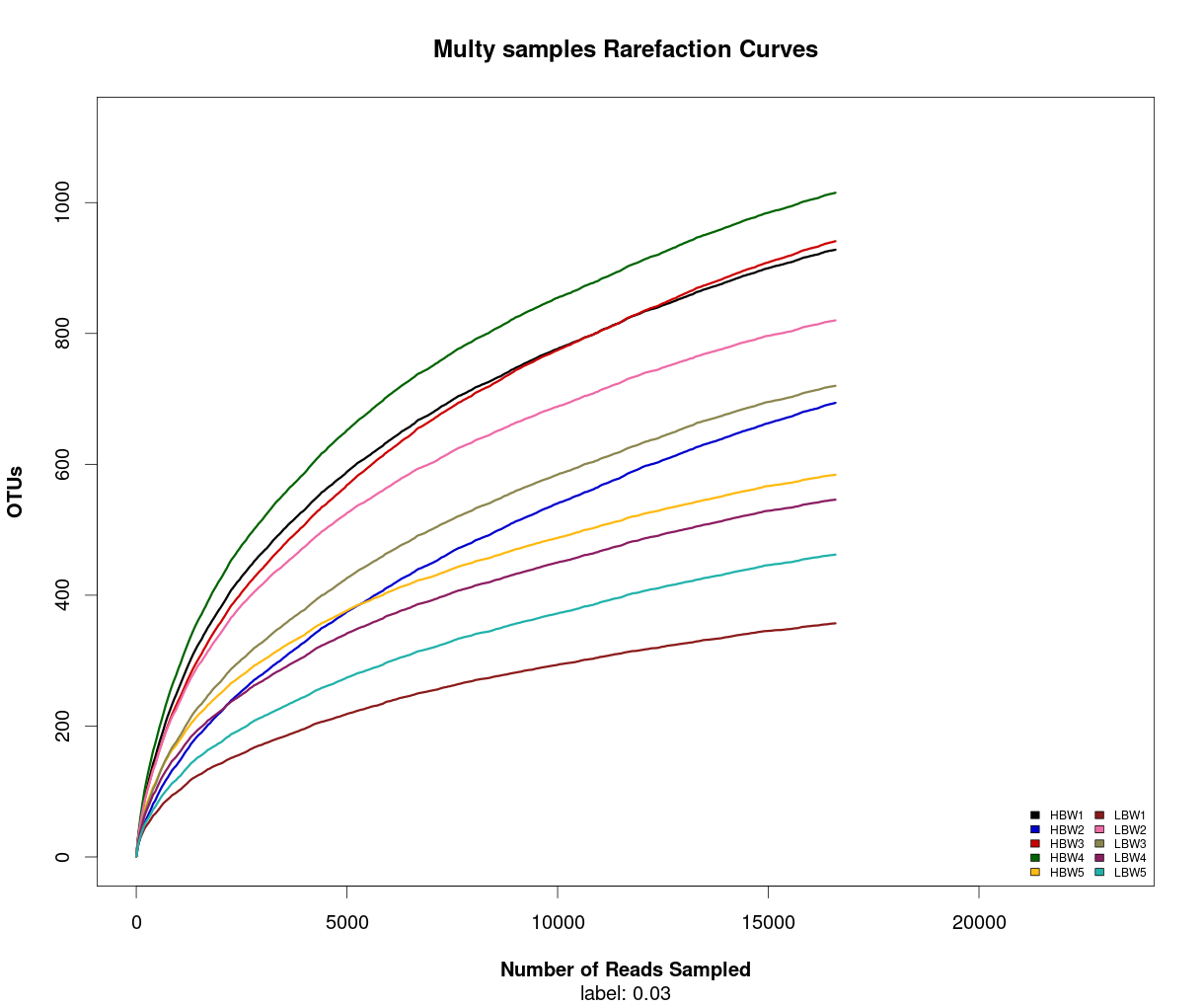

Supplement: Supplementary file 1 [file animals-14-00425-s001.zip › Supplementary Figure S2..png]

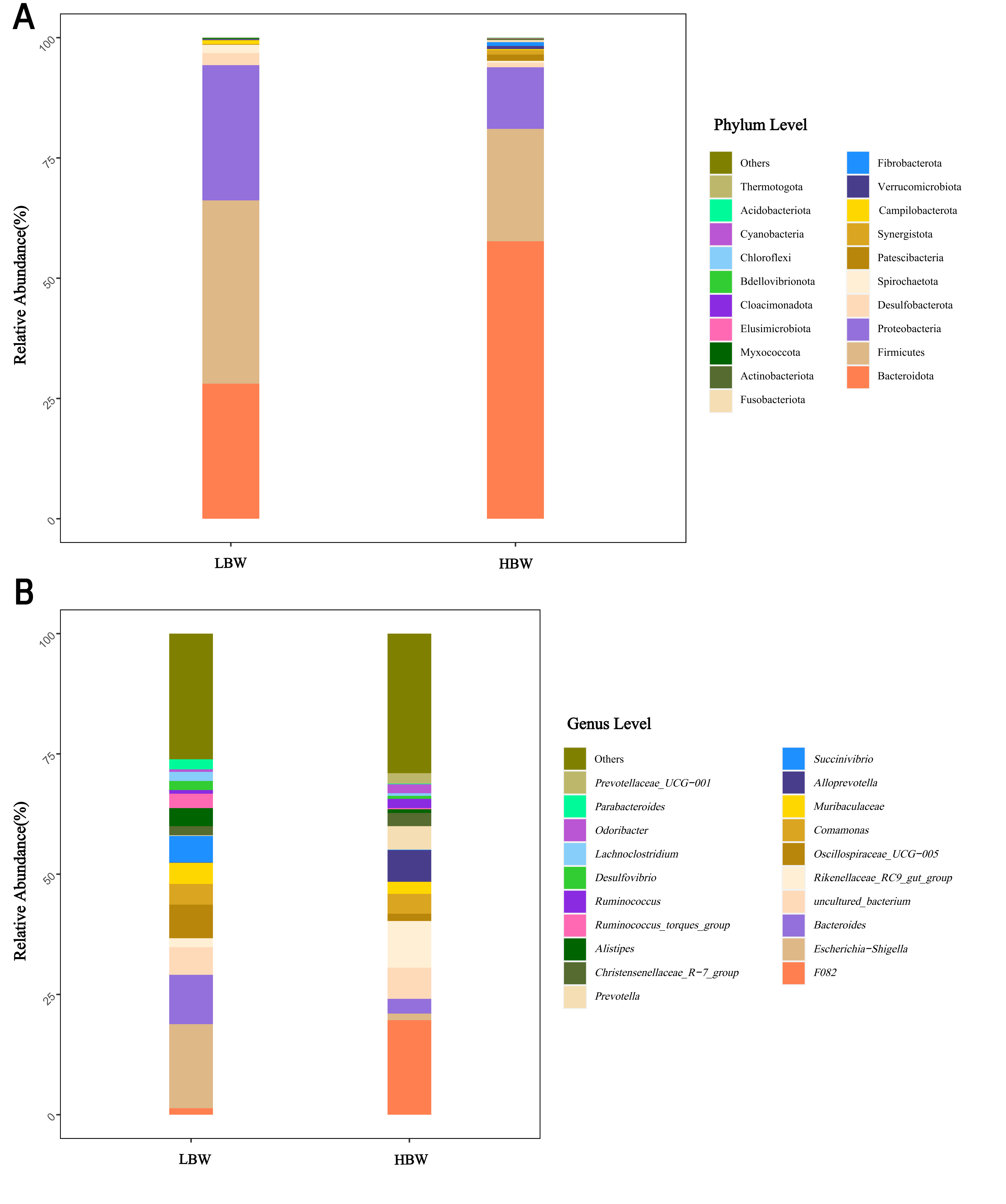

Supplement: Supplementary file 1 [file animals-14-00425-s001.zip › Supplementary Figure S3..png]

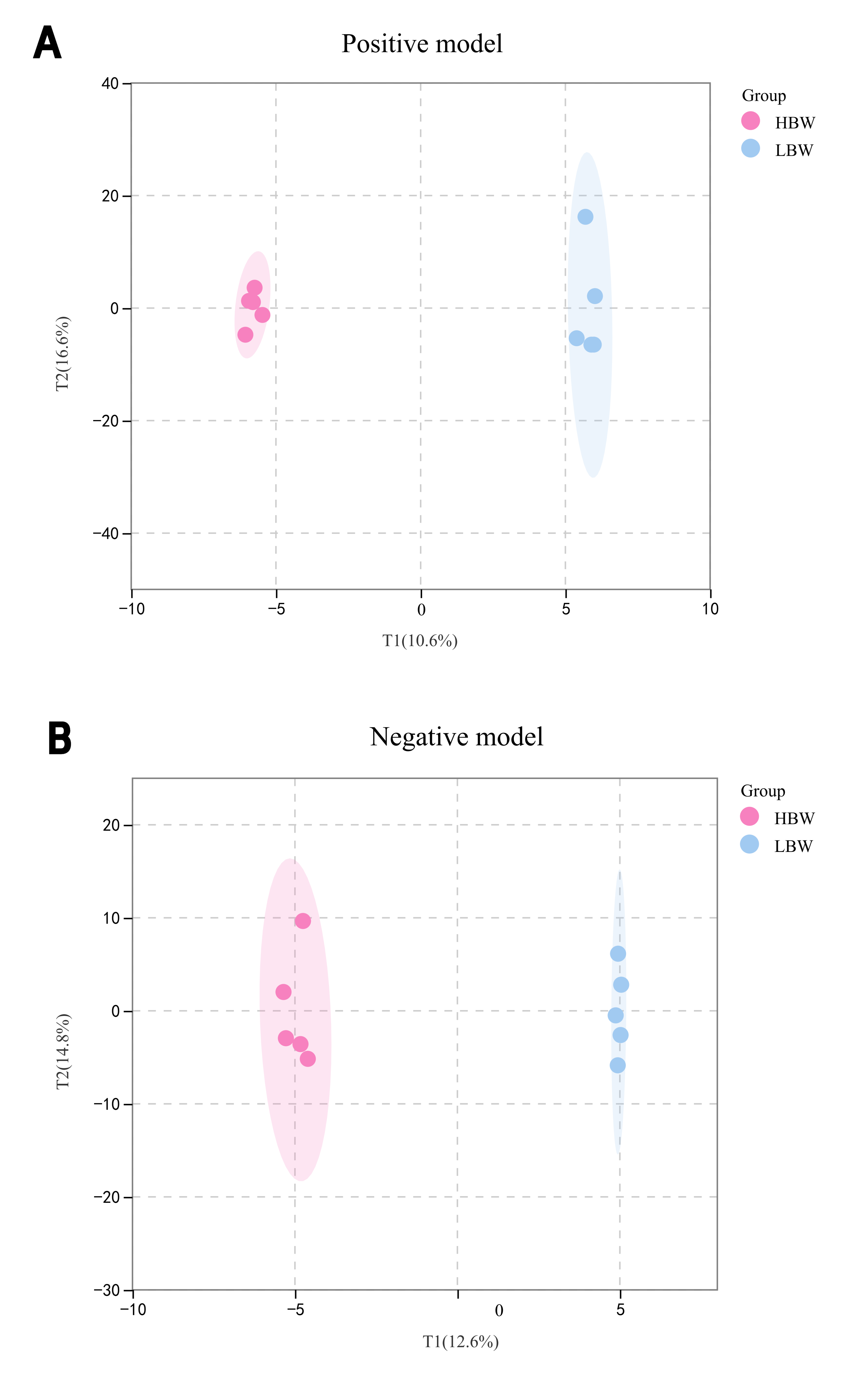

Supplement: Supplementary file 1 [file animals-14-00425-s001.zip › Supplementary Figure S4..png]
